# Readmission Rates of Patients with Schizophrenia Treated with Depot Antipsychotics versus Oral Antipsychotics in a Community Hospital

**DOI:** 10.7759/cureus.3775

**Published:** 2018-12-24

**Authors:** Eduardo D Espiridion, Callum Lewandrowski, Shiva Shahriari, Dorothy F Bestoyong

**Affiliations:** 1 Psychiatry, Frederick Memorial Hospital, Frederick, USA; 2 Osteopathy, West Virginia School of Osteopathic Medicine, Lewisburg, USA

**Keywords:** schizophrenia, readmission rates, depot antipsychotics

## Abstract

This is a pilot study comparing the 30 and 60 day relapse rates for schizophrenic patients treated with long-acting injectable antipsychotics versus traditional oral antipsychotics in a community hospital setting. This is a retrospective cohort study and medical records were reviewed to gather data involving readmission rates. We analyzed the data using relative risk (RR) for the 30 days and 60 days after patient discharge from the behavioral health unit of a community hospital.

## Introduction

Schizophrenia is a serious and chronic psychiatric condition characterized by delusions, hallucinations, disorganized behaviors, and social withdrawal. There are problems associated with treatment compliance because of poor insight and impaired judgment. The compliance issue leads to worsening clinical symptoms and psychiatric hospitalizations. The Centers for Medicare and Medicaid Services has identified readmission rate as an important quality marker in patient care [1]. It is one factor that affects the benchmark quality markers of inpatient behavioral health programs nationally. Finding ways to improve patient care is the goal of both hospitals and psychiatrists. This study aimed to determine if using depot antipsychotics versus oral antipsychotics improved the readmission rates in a behavioral health unit of a community hospital.

## Materials and methods

This pilot study was performed as a retrospective cohort analysis of schizophrenic patients treated in a 21-bed behavioral health unit of a community hospital. Subjects who were diagnosed with schizophrenia and treated as inpatients in the behavioral health unit of a community hospital between January 2013 to March 2018 met the inclusion criteria. We reviewed the medical records of patients with schizophrenia and bipolar disorder, who were ages 18 to 64 years. Patients with a diagnosis of bipolar disorder were included to determine if they were eventually diagnosed with schizophrenia. Only patients with a diagnosis of schizophrenia were included in the study. The information gathered from the medical records included demographic information, medication lists, hospital visits, and aftercare information. In addition, they must have been treated during this admission with either oral or long-acting injectable antipsychotics.

Patients were excluded from the study if they were diagnosed with a mental illness other than schizophrenia and this included substance-induced psychosis. Also, patients with schizophrenia who were not treated with antipsychotic medication and patients with schizophrenia on more than one oral antipsychotic medication at the time of discharge from the inpatient psychiatry unit were excluded. Finally, patients with schizophrenia on a depot antipsychotic medication with another oral antipsychotic medication as maintenance treatment at the time of discharge were excluded as well.

There were 278 patients who met these criteria, and 178 were male and 100 were female. These patients were divided into two groups, those treated with oral antipsychotics (193) and those treated with long-acting injectable antipsychotics (85). The top three oral antipsychotics were olanzapine, risperidone, and quetiapine. The top three long-acting antipsychotics used paliperidone palmitate extended release (69), aripiprazole extended release injectable (14), and haloperidol decanoate (2).

We then investigated the number of behavioral health unit readmissions among these patients at both 30 and 60 days. Emergency department and other non-inpatient behavioral health visits were not included, unless they led to a behavioral health unit admission. The number of readmissions for the long-acting injectable group was compared to that of the oral antipsychotic group. The relative risk and number to treat were all calculated for both 30 and 60 day readmissions.

## Results

Among the patients treated with oral antipsychotics, 29 (15.0%) relapsed within 30 days. An additional 24 (12.4%) patients relapsed between 30 and 60 days, making a total of 53 (27.5%) 60 day relapses. For the 85 patients treated with the long-acting depot antipsychotics, seven (8.2%) relapsed within 30 days. Another seven (8.2%) relapsed between 30 and 60 days, making a total of 14 (16.5%) 60 day relapses. Using this data, we calculated an absolute risk reduction for long-acting antipsychotics versus oral antipsychotics of 6.8% at 30 days and 11% at 60 days. The relative risk between the two groups at 30 days was calculated to be 0.55 with a 95% confidence interval of 0.25-1.20 (p+0.133). At 60 days, the relative risk was calculated to be 0.60 with a confidence interval of 0.35-1.01 (p=0.059). The number to treat was calculated at 14.7 for 30 days and 9.1 for 60 days.

## Discussion

This retrospective cohort study determined a reduction in the readmission rates of the sample population. However, because of the small sample size the null hypothesis cannot be confidently rejected. The results of the study did show a 7% and 11% decrease in the 30 day and 60 day readmissions. This finding will lead to further discussion or discourse into a deeper and larger scale research on depot antipsychotics versus oral antipsychotics to possibly demonstrate a statistically significant result.

When looking at larger studies, there seems to be a mixed conclusion on the efficacy of antipsychotics involving the long-acting depot antipsychotics compared to the oral antipsychotics. Specifically, two large scale meta-analyses looked specifically at patient relapse rates with the oral antipsychotics and depot antipsychotics. The first study was a 10 study meta-analysis that looked at a minimum of 12-month relapse rates. There were a total of 1700 participants in an inpatient setting and it showed a 30% absolute risk reduction [2]. The second study was done as a follow-up to the first study mentioned. It was the largest meta-analysis that compared depot antipsychotics versus oral antipsychotics. It was based on 21 randomized clinical trials with 5176 participants. Patients were enrolled from both the inpatient and outpatient settings. The study did not show any evidence of superiority with the depot antipsychotics or oral antipsychotics in improving the relapse rates [3].

In this study, we included all patients with a diagnosis of schizophrenia who received pharmacological treatment with antipsychotics. We looked at patients who were initially diagnosed with bipolar disorder and had a diagnosis of schizophrenia on discharge. A two-year study that looked at first episodes of psychosis and compared the relapse rates of patients who received oral risperidone versus depot risperidone showed that risperidone long-acting injectables significantly reduced the relapse rates when compared to oral risperidone [4]. Additionally, patients who had a first episode of psychosis and who received the depot risperidone had a two-thirds decrease in hospitalization rates compared to those who were treated with oral risperidone [5]. Among the general population of patients with schizophrenia, there is evidence that patients treated with haloperidol or fluphenazine injectables had a significantly longer mean time to all cause medication discontinuation compared to patients treated with oral haloperidol or fluphenazine [6].

We reviewed only the patients in the inpatient behavioral health unit with schizophrenia, who received antipsychotic medications. The sample size would be increased if we also looked at the outpatient settings. A meta-analysis of patients with schizophrenia in both inpatient and outpatient settings found an interesting point of bias. The patient population showed increased efficacy differences between the depot antipsychotics and oral antipsychotics in a randomized study of schizophrenia [7].

There have been conflicting results from research studies involving the efficacy as well as readmission rates of schizophrenia patients using long-acting depot antipsychotics versus oral antipsychotics. Patients in the outpatient setting have issues with noncompliance leading to an underestimation of the true efficacy of the depot antipsychotics. Compliance is a significant issue in healthcare, and it is truly a problem among patients with schizophrenia. Patients in this community hospital received aftercare wraparound services with case management. This has positively impacted readmission rates. Patients who kept their scheduled appointments received their medications in a timely manner. There were issues with tolerability and its impact on compliance in both treatment groups. Patients who experience an adverse event will likely not comply with treatment follow-ups. This leads to an exacerbation of psychotic symptoms and readmissions.

## Conclusions

As this was a pilot study, our sample size was too small to draw any conclusions. However, the data suggests that the relapse rates may be better with long-acting depot antipsychotics. A bigger sample size is needed to obtain statistically significant results. We recommend further studies with larger sample sizes to determine if long-acting depot antipsychotics reduce the 30 and 60 day relapse rates in the community hospital setting.
